# Treatment Outcome and Prognostic Factors of Malignant Thymoma - A Single Institution Experience

**DOI:** 10.31557/APJCP.2020.21.3.653

**Published:** 2020-03

**Authors:** Haya S Alothaimeen, Muhammad A Memon

**Affiliations:** 1 *Oncology Center, King Faisal Specialist Hospital and Research Centre, Riyadh, Saudi Arabia, *; 2 *Cancer Institute, Geisinger Health System, Pennsylvania, USA.*

**Keywords:** Thymoma, thymic carcinoma, treatment modality, prognostic factors

## Abstract

**Objective:**

Our objectives are to investigate the clinicopathological features, treatment modalities, and prognostic and prognostic factors in order to estimate long-term outcomes for patients with thymoma and thymic carcinoma at our institution.

**Methods:**

We reviewed all patients diagnosed with thymic malignancies malignancies over a period of 38 years (from 1976 to 2014). Patients were identified using a single institution database at King Faisal Specialist Hospital and Research Center (KFSH and RC), Riyadh. Demographic data, clinical staging, histopathology classification, treatment approaches, and survival data were collected. Data Analysis was performed using both the Kaplan–Meier method and Cox proportional hazards modeling.

**Results:**

The fifty-six identified patients consists of 30 females (53.6%) and 26 males (46.4%). The median age at diagnosis was 39 years. About 37% of the patients were diagnosed with myasthenia gravis (MG). There was a significant association between the WHO histologic classification and the Masaoka stage (p= 0.018). The estimated 5-year overall survival rate was 88.6% for patients with thymic malignancies. The median survival time of thymoma and thymic carcinoma was 61 and 14 months, respectively. The univariate analysis suggested that histology (thymoma versus thymic carcinoma, p= 0.044) and Masaoka stage (II-III versus IV, p= 0.048) were independent prognostic factors affecting overall survival. Histology (p = 0.044) was found to be an independent predictor of overall survival.

**Conclusion:**

The findings of this study indicates that late Masaoka-Koga staging and histology types are significantly associated with extended overall survival. Similarly, surgical resection and multimodality treatments play a significant role in thymic malignancies neoplasms therapy strategies to prolong survival rates.

## Introduction

Thymoma and thymic carcinoma are rare neoplasm types that originate in the thymus gland (Modh et al., 2016). The tumor cells in thymoma have indolent growth behavior (Rieda et al., 2016). Histologically thymomas appear as benign tumors, even though they may exhibit malignant behavior (Detterbeck and Zeeshan, 2013). Thymomas is the most common neoplasm of the anterior mediastinum, accounting for 20-25% of all mediastinal tumors and 50% the anterior mediastinum is thymoma, (Venuta et al., 2011). On the other hand, the tumor cells in thymic carcinoma are more aggressive and it tends to spread to other parts of the body (Levine and Rosai, 1978). Thymic carcinomas represent 15% to 20% of all thymic neoplasms (Venuta et al., 2010). 

The World Health Organization (WHO) has developed a classification system based on the cells that make up the tumor for thymoma. This classification distinguishes thymomas (Thymoma subtypes A, AB, B1, B2, and B3) from thymic carcinoma (Type C) (Marx et al., 2014). The staging has been proposed by Masaoka et al., (2010) in 1981.

Many studies report that the risk factors and etiologies of thymoma and thymic carcinoma not well addressed due to the rarity of the tumors (Rieda et al., 2016; Travis et al., 2015; Kim et al., 2001; Carter et al., 2014). People with thymoma may develop other autoimmune diseases such as Myasthenia gravis (MG); identified as an autoimmune disorder that causes muscle weakness, present in 30% of patients with with thymoma. Only 10 to 15% of patients of patients with MG also develop thymoma (Tomaszek et al., 2009). There are no available screening tests for thymoma/thymic carcinoma; about one-third of the incidents diagnosed and discovered accidentally (Marom et al., 2011; Girard et al., 2015).

Thymic malignancies are usually diagnosed, staged, and treated during surgery. The best treatment approaches of thymic malignancies tumors remain debatable among physicians. Previous studies have determined that patients with thymic carcinoma have a worse prognosis compared to thymomas patients (Engels and Pfeiffer, 2003; Gripp et al., 1998; Engels, 2010). Complete surgical resection is considered the treatment of choice for patients with thymoma/thymic carcinoma in early stages, while radiotherapy and/or chemotherapy have been suggested for advanced and metastatic stages (Kondo and Monden, 2003). Many studies imply that the prognostic factors in clinical parameters are disease stage, tumor resection status, histological tumor classification, and patient’s performance status (Marx et al., 2014; Tomaszek et al., 2009; Engels, 2010).

Identifying the critical therapy courses that impact prognosis positively from the currently available therapies that improve the response rate will help us to provide arguments for etiologic research. Because of their indolent course, diagnosis and therapy approaches have mainly been based on retrospective or descriptive epidemiological studies of thymic malignancies. Limited epidemiological studies have been focused on long-term outcomes and treatment modality in the Middle East, where the disease tends to be more prevalent (Ahmed et al., 1995; Engels, 2010; Weis et al., 2015; Van Kolen et al., 2010). The objectives of this study were to investigate the clinicopathological features, treatment modalities, and prognostic factors in order to estimate long-term outcomes for patients with thymoma and thymic carcinoma, and who have been treated at our institution over 38 years.

## Materials and Methods

From January 1976 to December 2014, a total of 59 patients diagnosed with thymic malignancies tumors treated in the oncology department at King Faisal Specialist Hospital and Research Centre (KFSH and RC), Riyadh, Saudi Arabia. We retrospectively reviewed the medical records after after obtaining ethics approval from the Institutional Review Board (IRB). Out of 59, three of them were excluded due to insufficient medical record data. Therefore, 56 patients were included in of this study. Relevant demographic information, clinical staging data, histopathology classification data, treatment approaches data, and overall survival, were reviewed and analyzed.

*Statistical Analysis*

Data analysis was conducted using the Statistical Package for Social Sciences (SPSS) software, version 24.0 (IBM Corp., Armonk, N.Y., USA), and JMP Pro 13 (SAS Institute, Cary, NC, USA). Continuous variables were quoted as median, mean and interquartile ranges. Statistical differences between patients’ groups were determined by chi-square, likelihood ratio test, and fisher’s exact test. The level p<0.05 was considered as the cut-off value for significance. 

Overall survival (OS) was defined as the time between treatment or last follow-up and death from any cause. Disease-free survival (DFS) was defined as the time from initial treatment date to the first evidence of recurrent disease death from disease, or last follow-up. Survival analysis was undertaken using the Kaplan-Meier method for actuarial survival (Kaplan and Meier, 1958). Differences between survival rates were tested using the log-rank test. Cox’s proportional hazard model was used to assess the independent significance of each risk factor (Prentice, 1992). Hazard ratios (HRs) and the 95% confidence intervals (95% CIs) were provided for each model. The variables that were analyzed included age (equal or greater than 45 versus less than 45 years), gender (male vs. female), associated with Myasthenia gravis (MG), Masaoka stage (II-III vs. IV), histology types (A-B3 types vs Type C), resection status (complete vs. incomplete), treatment modality (multimodal vs. bi- or single modality), and recurrence (Yes vs. No).

## Results

Fifty-Nine cases of thymic malignancies found in the tumor registry, three of them were excluded due to insufficient medical record data. The results of this study were obtained form to total of 56 patients. Relevant demographic information, clinical staging data, histopathology classification data, treatment approaches data, and overall survival, were analyzed and the findings will be presented and discussed in the following section.

*Demographics*

Eighty percent (80%) of the patients were pathologically confirmed with malignant Thymoma, and 20% of them were thymic carcinomas (TCs). The demographic distribution of the population is described in Table 1. The median age at diagnosis was 39 years, and the overall mean ± SD was 40 ± 17 years; range of 13 – 86. The higher proportion of patients (37%) at diagnosis were aged 18 to 35 years old) and only 3 patients were under the age of 18 years. The gender composition of this study was 53.6% male and 46.4% female; with no family reported cases. Table 1 indicates that 87.5% of the patients were Saudis, and 12.5% were non-Saudi patients; they were from Thailand, Sudan, Yemen, Egypt, Syrian, and Philippine. 

*Clinical Presentation and Diagnostic Method*

Fifty-four patients were presented with symptoms dyspnea (67.86%), cough (48.22%), chest pain (48.21%), and muscle weakness 10 (17.86%). Twenty patients (35.7%) had myasthenia gravis. During the follow-up period, Result shows that the majority of the patients had a biopsy (89.3%), followed by CT scans, chest X-ray, and PET scans (87.5%, 44.6%, 32.1%, retrospectively). All related results are shown in Table 1. 

*Histopathology Classification and Staging*

**Figure 1:**
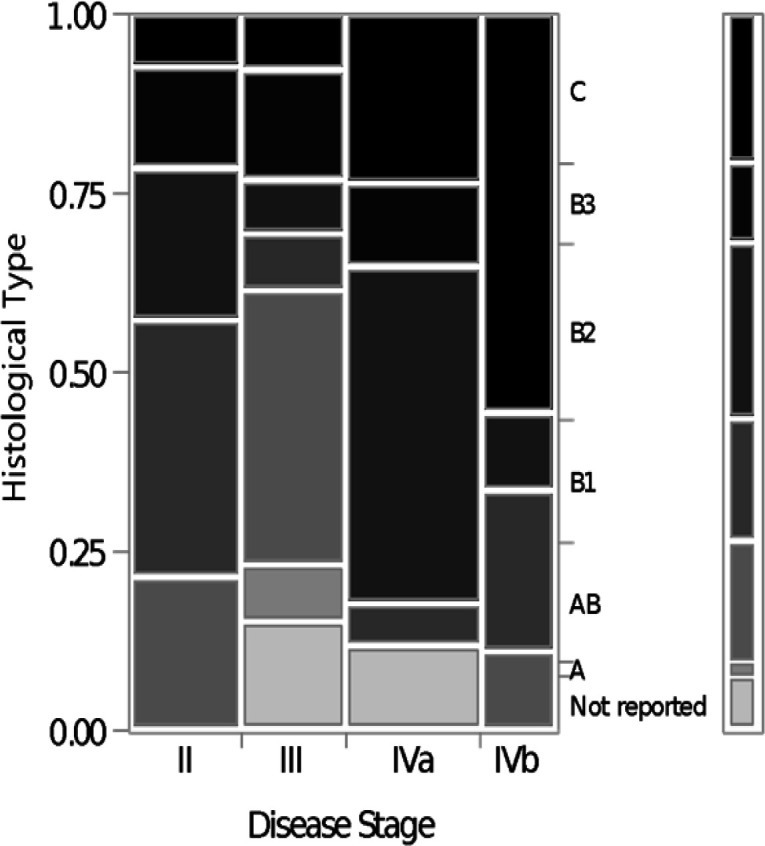
Shows the Association between Masaoka-Koga Stage and WHO-Based Histology Classification. (Likelihood Ratio, P=0.0077)

The distribution of histologic subtype and Masaoka staging system is provided in Table 1. Thus, none of the patients was diagnosed with stage I, well-known as as a benign tumor, in our patient population with thymic malignancies tumor. There were 3 patients without stage preference and 10 patients without WHO histologic classification. The relationship between WHO classification and Masaoka staging system is shown in Figure 1. There was a significant association between WHO classification types and the Masaoka staging (Likelihood Ratio, p = 0.007). 

*Treatment modality*

Twenty-four patients (42.8%) received multimodal therapy (surgery, chemotherapy, and radiation therapy), 23 patients (41.1%) had bimodality treatment, and only 9 patients (16.1%) underwent unimodality treatment. Unrespectable tumors frequently were seen in patients diagnosed with malignant thymoma advanced stages. Surgical procedures consisted of either total or subtotal thymectomy through median sternotomy, or debulking surgery. The treatment modalities offered and tumor resection status distribution for all patients is indicated in Table 2. 

*Survival Analysis*

Twenty-one patients (39%) had a recurrence; 17 (80.9%) developed metastatic recurrence, and 4 (19%) patients developed local and metastatic recurrence. The median time follow-up for all patients at the time of the analysis was 65 months (range: 0 – 243 months, 95% CI: 45-91), 34 patients (60.6%) were alive and without recurrence, 14 (25%) were alive and without recurrence 8 (14%) died due to the disease.

**Figure 2:**
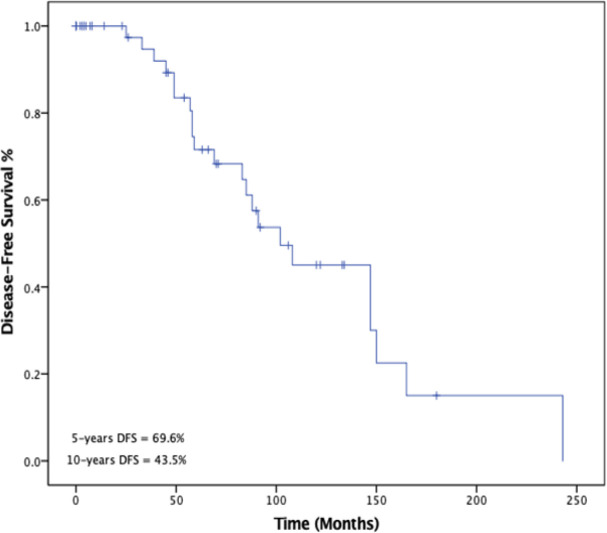
Kaplan-Meir Survival Curve of 5- and 10-Years Disease-Free Survival (DFS)

**Figure 3:**
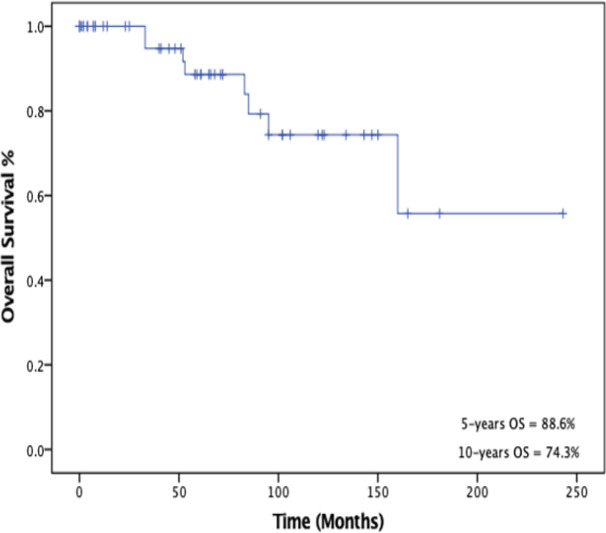
Kaplan-Meier Survival Curve of 5- and 10-Years Overall Survival (OS)

Survival analysis was performed to measure overall survival (OS) and disease-free survival (DFS) as outcomes. The 5-year and 10-year disease-free survival (DFS) rates were 69.6% and 43.8% %, respectively as shown in Figure 2. The 5- and 10-years overall survival (OS) rates were 88.6% and 74.3%, respectively as illustrated in Figure 3. Five-year survival rate for thymoma and thymic carcinoma were 90.1% and 75%, respectively. There was a statistically significant difference (p = 0.0281) between these two groups when compared using log-rank test.

As for Masaoka staging, 5-years OS rates with regards to stage II, III, and IV were 87.5%, 100%, and 80.2%, respectively (log-rank test, p = 0.16), and 5-years DFS rates were were 87.5%, 75%, and 55% respectively (log-rank test, p = 0.09). For WHO histology subtype, 5-year survival rates in A, AB, B1, B2, B3, and C types types were 90%, 100%, 75%, 100%, 100% and 75%, respectively (log-rank test, p = 0.29). With DFS, survival rate differences among the five histologic types were 100%, 83.3%, 75%, 63.3%, 67% and 80% respectively, (log-rank test, p = 0.96). With regards to treatment modality the 5- and -10-year survival rate of patients with multimodal therapy were 84.2% and 65.3%, respectively (log-rank test, p = 0.06). For resection status, the 5- and 10-year survival for patients with complete resection was higher than those patients with partial resection (81% and 81%, 100% and 50%, respectively). There was a significant difference among patients who underwent complete resection of malignant thymoma and thymic carcinoma (log-rank, p = 0.0001). Moreover, for these two groups of patients there was a significant difference among patients who underwent partial resection when the same comparison was performed (Log-rank test: p = 0.0177).

*Recurrence Pattrens*

Twenty-one patients (39%) had a recurrence, (average recurrence of 3.9 after treatment; median of 3 years, range 2 - 11 years). The median recurrence-free survival for all patients was 102 months (95% CI, 69-147). Seventeen had metastatic recurrence, and 4 patients had both local and metastatic recurrence. The 2-year survival rate of the patients with recurrent disease was 90.9%, with a median survival period of 38 months. Disease-related death occurred in eight patients. Of note, the result observed that only one patient who underwent chemotherapy followed by tumor resection has more than one recurrence during this period. 

Ten of the patients who had recurrence treated with surgery and postoperative radiotherapy or chemotherapy.

Eighteen (40%) of malignant thymoma patients had a disease recurrence the 3- and 5-year Recurrence-free survival (RFS) was 97% and 71%, respectively. The most recurrent patterns related to the disease stages were stage II 3- and 5 year RFS with 100% and 88%, respectively. Stage III had a RFS similar to Stage II (100%, 75%). whereas, 83% of patients with stage IVa and 48 % had a disease recurrence after 3 years and after 5 years, respectively. Stage IVb had higher disease recurrence in 5 years compared to stage IVa (60%). 

*Prognostic Factors*

We analyzed several predictor factors including age, present of Myasthenia gravis, stage, histotype, resection status, treatment modality, and recurrence utilizing overall survivalas shown in Table 3. The univariate analysis demonstrated that; histology and advance stage yields a worse prognosis. Histology types and stage were also tested using the Cox proportional hazards model. With multivariate analysis, only histology (thymoma versus thymic carcinoma) showed significant independent effects on overall survival. No significant predictors of disease recurrence were observed using univariate and multivariate analysis for disease-free survival (DFS).

**Table 1:** Distribution of Demographic and Clinical Characteristics among All Patients, Thymoma Patients, and Thymic Carcinoma Patients

Characteristics	Thymoma (n= 45, 80.36%)		Thymic Carcinoma (n=11, 19.64%)		Overall (n=56)	
	N	%	N	%	N	%
Age at Diagnosis						
Median Years	39		40		39	
Range	13-86		16-63		13-86	
Age Group						
< 18	2 (3.57)		1 (1.79)		3 (5.36)	
18-35	17 (30.36)		4 (7.14)		21 (37.50)	
36-55	16 (28.57)		2 (3.57)		18 (32.14)	
55 <	10 (17.86)		4 (7.14)		14 (25)	
Gender						
Female	22	39.29	4	7.14	26	46.43
Male	23	41.07	7	12.5	30	53.57
Nationality						
Saudi	6	10.71	1	1.79	7	12.5
Non-Saudi	39	69.64	10	17.86	49	87.5
Symptoms						
Dyspnea	31	55.36	7	12.50	38	67.86
Cough	22	39.29	5	8.93	27	48.22
Chest Pain	20	35.71	7	12.5	27	48.21
Muscle Weakness	8	14.29	2	3.57	10	17.86
Diagnostic Methods						
CT	38	67.86	11	19.64	49	87.5
Chest X-ray	18	32.14	7	12.5	25	44.64
PET scan	16	28.57	2	3.57	18	32.14
WHO Histology Classification (P= 0.0001)						
A	1	1.79	0	0.00	1	1.79
AB	9	16.07	0	0.00	9	16.07
B1	8	14.29	0	0.00	8	14.29
B2	12	21.43	0	0.00	12	21.43
B3	5	8.93	0	0.00	5	8.93
C	0	0.00	11	19.64	11	19.64
Missing	10	17.86	0	0.00	10	17.86
Masaoka Staging System (P= 0.035)						
II	13	23.21	1	1.79	14	25.00
III	12	21.43	1	1.79	13	23.22
IVa	13	23.21	4	7.14	17	30.35
IVb	4	7.14	5	8.93	9	16.07
Missing	3	5.36	0	0.00	3	5.36
Myasthenia gravis (MG)	18	32.14	2	3.57	20	35.71
Smoking	9	16.07	3	5.36	12	21.43
Recurrence	18	32.14	4	7.14	22	39.28

**Table 2:** The Distributing of Treatment Characteristic among All Patients

Characteristics	Thymoma (n= 45, 80.36%)		Thymic Carcinoma (n=11, 19.64%)		Overall (n=56)	
	N	%	N	%	N	%
Treatment Modality						
Tri-modality	7	12.50	2	3.57	9	16.07
Bimodality	18	32.14	5	8.93	23	42.86
Single-modality	20	35.71	4	7.14	24	42.86
Treatment Received						
Surgery only	4	7.14	1	1.79	5	8.93
Surgery and Radiotherapy only	13	23.21	3	5.36	16	28.57
Surgery and Chemotherapy only	2	3.57	1	1.79	3	5.36
Surgical Resection Status						
Complete resection	21	37.50	2	3.57	23	41.07
Partial resection	11	19.64	2	3.57	13	23.21
Simple biopsy	6	10.71	4	7.14	10	17.86
Inoperable tumor	7	12.50	3	5.36	10	17.86

**Table 3:** Cox Proportional Hazards Analysis of Overall Survival for Both Thymoma and Thymic Carcinoma Patients

	Univariate			Multivariate		
Indicators Variables	Hazard Ratio (Exp B)	95% CI	P-value	Hazard Ratio (Exp B)	95% CI	P-value
Age (y)						
> 45	0.024	0.00 - 11.20	0.233	_	_	_
≤ 45						
Gender						
Female	4.260	0.84 - 12.56	0.800	_	_	_
Male						
Present with MG						
Yes	0.436	0.08 -2.17	0.310	_	_	_
No						
Stage						
I-III	4.668	1.01-21.44	0.048*	0.31	0.05 - 1.70	0.179
IVa-IVb						
Histology						
Thymoma	4.502	1.41-19.47	0.044*	0.22	0.05 - 0.96	0.044*
Thymic-Carcinoma						
Resection						
Complete resection	0.623	0.14-2.6	0.517	_	_	_
Uncompleted resection						
Treatment Modality						
Trimodality	0.172	0.02-1.44	0.105	_	_	_
Bi/Single modality						
Initial treatment						
Surgery	0.334	0.07-1.43	0.140	_	_	_
Non-Surgery						
Initial treatment						
Chemotherapy	3.654	0.85 - 15.7	0.082	_	_	_
Non- Chemotherapy						
Recurrence						
Yes	4.998	0.61-40.84	0.133	_	_	_
No						

*The Chi-square statistic is significant at the 0.05 level.

**Figure 4:**
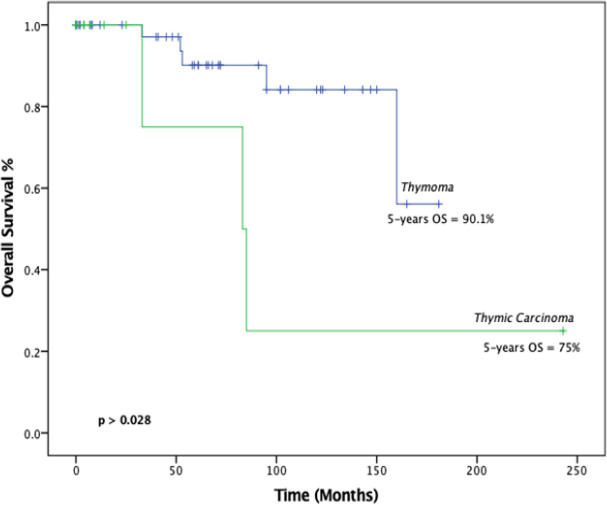
Kaplan-Meier survival analysis of overall survival classified according to disease histologic diagnosis thymoma and thymic carcinoma. The curve showing median overall survival of thymoma patients was 61 months, and for thymic carcinoma 84 months (95% CI, 160-not reaches) (P < 0.05). The 5- and 10-year overall survival for Thymoma was 90% and 84%, while thymic carcinoma was 75% and 25%, respectively

## Discussion

Patients demographic, clinical presentation, Masaoka-Koga stage, WHO histologic subtypes, and treatment modality were investigated in this study identify the long-term outcome, and to look at the prognostic factors that might affect patients’ survival. The findings from this study indicate the following associations: (i) a Masaoka-Koga staging system correlates with WHO histologic subtypes significantly; (ii) among the variables of interest, Masaoka-Koga stage and histologic types were the only prognostic factor in univariate analysis; and (iii) histologic classification was the only independent prognostic factor factor based on the multivariate regression model. 

Thymoma and thymic carcinoma incidence rise in middle aged patients with no gender differentiation. In this patient population, thymoma is more common in patients older than 30 years and years, which is earlier than the reported in previous studies (Engels and Pfeiffer, 2003; Okuma et al., 2013; Moore et al., 2001). Thymic malignancies incidence are not common in children, only one female aged 13 years old was diagnosed with malignant thymoma. Age is considered a vital feature to evaluate patients with thymic malignancies tumors since some of the anterior mediastinal mass tends to be more common in a certain age age in agreement with published literature. There is no gender preference in thymic malignancies tumors. The gender composition of this study was similar to to studies published in references (Ruffini et al., 2014; Girard et al., 2015; Kondo and Monden, 2003). The most frequent symptoms presented in patient population were dyspnea (68%), cough (48%), chest pain (48%), and myasthenia gravis (36%) (Ruffini et al., 2014; Kondo and Monden, 2003; Pescarmona et al., 1990). Unlike previous studies that addressed myasthenia gravis role as an adverse factor in survival (Lucchi et al, 2009; Legg and Brady, 1965), our results indicate that the presence of MG has no impact on survival and/or recurrence. 

Different diagnostic tests were applied to classify the the types of tumors, not all lesions in the anterior mediastinum are biopsied. CT scan was usually the initial imaging modality used to evaluate thymic malignancies tumors (Carter et al., 2014). A previous study stated that CT is important in predicting whether a tumor is easily resectable, and useful for detecting recurrence after a prior resection (Detterbeck and Zeeshan, 2013; Blumberg et al., 1995). There was no significant difference observed on long-term survival between CT and biopsy. In this study, malignant thymoma accounts for more than two-thirds (2/3) of the patient population and only 20% were thymic carcinoma, while the majority were metastatic tumors.

In the present study, the overall survival rates of thymoma and thymic carcinomas were similar to other studies, which indicate they have a good prognosis compared to other types of cancers. In our analysis, we investigated the long-term outcome using the Masaoka-Koga staging system, and WHO histology subtype classification. We observed a significant association between staging and histology classification which is coherent with the literature (Okuma et al., 2013; Rena et al., 2005; Tomiyama et al., 2002; Weis et al., 2015; Safieddine et al., 2014). However, our findings demonstrate that stage II and stage III have better survival and recurrence-free compare to other stages. In contrast, stage IV showed significantly worse survival and recurrence. According to WHO classification, our results reveal that thymomas type AB showed remarkable survival. Our population outcomes based on stage and histology subtypes provided different insights compared to some previous studies (Wilkins, 1991; Huang et al., 2009; Regnard et al., 1996). Pathology results were unavailable due to the rarity of the disease.

In this study, there was a variation in the treatment methods offered to thymoma and thymic carcinoma patients. Several studies have been performed to identify the most effective treatment strategy for thymic malignancies tumors (Blumberg et al., 1995; Regnard et al., 1996; Maggi et al., 1991). These studies showed that surgery is the best treatment option for patients with early-stage of thymic malignancies tumors and influence on long-term survival positively (Blumberg et al., 1995; Regnard et al., 1996; Ríos et al., 2002). Inadequate data of the radiotherapy and chemotherapy regimens/course due to the absence of documentation led us to focus on what what is available in the patient records such as treatment modality and resection status. Our study indicates a significant long-term survival of patients who underwent multimodal treatment (Kim et al., 2004; Spaggiari et al., 2012). Complete resection of the tumor remains the core of treatment for thymic malignancies tumors (Kondo and Monden, 2003; Rea et al., 2004; Lewis et al., 1987; Rieker et al., 2002). In our study, patients who underwent complete resection had better survival and recurrence rate than those who had a partial resection, simple biopsy, or inoperable tumor (Hsu et al., 2002; Magois et al., 2008; Ogawa et al., 2002). The few patients who had no therapeutic intervention will to be investigated in a separate study. In this retrospective study, we do not consider these results as certainly, multimodality treatment can be considered to patients with the advanced-stage disease. 

The behavior of thymic malignancies is is not well established compared to other types of cancers; the disease recurrence is a common feature and was analyzed separately. Numerous studies focus on overall survival as an endpoint, but limited studies focused on recurrence (Girard et al., 2015; Blumberg et al., 1995). Our results showed that recurrence occurred in 40% of thymomas, while 50% of the carcinomas had a recurrence. The recurrence rate of thymoma and thymic carcinomas varies according to different reports (Ruffini et al., 2014; Mizuno et al., 2015). Disease-free survival rates in our study were similar to previously published reports (Mariano et al., 2013; Luo et al., 2016). One interesting patient had metastatic thymoma disease at initial presentation and was treated with chemotherapy followed by radiotherapy and surgery. This patient had patient had a three recurrence in different time intervals and died with a thymoma-related cause. 

Thymomas and thymic carcinomas have indolent behavior and long-term survival. In this study, the median follow-up for all patients was 65 months similar to a previous study (Modh et al., 2016). In our analysis, the disease stage and initial histologic diagnosis (thymoma vs thymic carcinoma) were found the most significant predictors of overall survival (Wilkins, 1991). Similarly, the stage was also found to be a prognostic factor in other studies as well (Kim et al., 2001; Kondo and Monden, 2003; Rena et al., 2005). Unlike our study, prior studies have other factors that affect prognoses such as age, classification pattern, resection status, and age (Blumberg et al., 1995; Rena et al., 2005; Okuma et al., 2015). Based on our literature search there are limited cohort studies that include a large number of thymic malignancies because of their rarity and their indolent behavior.

As any retrospective study, there are several limitations, including the rarity of the disease, lack of documentation, and inclusion criteria due to the fact that the cancer registry is limited to malignant cases. As noted earlier, this is a single center study in the Middle East, hence the results cannot be generalized and further studies on larger patient populations are required to confirm and/or provide more insights of the findings of this study. 

We suggest that a focus group of the patients who are still alive from the present study, should be followed-up and interviewed for better understand the nature and the progression of these type of diseases. Alternatively, multicenter clinical trials in Asia with a larger number of patients are needed to assess overall survival in malignant Thymoma and Thymic malignancies. 

In conclusion, thymoma and thymic carcinoma are frequently present at advanced stages. Late Masaoka-Koga staging and histology types are significantly associated with extended overall survival. Multimodality and surgical resection play significant strategies to treat thymic malignancies tumors with excellent 5 and 10-year survival rates. Prospective randomized studies to establish an optimal treatment protocol are necessary for thymoma and thymic carcinoma patients.

## Declaration

The work was done under the guidance of Dr. Edward De Vol, Dr. Mohammed Memon and Dr. Fazal Hussain at King Faisal Specialist Hospital and Research Centre (KFSH&RC), Riyadh, Saudi Arabia.
